# Predictors of 30-Day Unplanned Readmission in Necrotizing Pancreatitis: A 12-Year Experience From a Tertiary Care Center

**DOI:** 10.14309/ctg.0000000000000848

**Published:** 2025-05-23

**Authors:** Gaurav Suryawanshi, Megan B. Ghai, Nauroze Faizi, Stuart K. Amateau, Nabeel Azeem, Shawn Mallery, Martin L. Freeman, Guru Trikudanathan

**Affiliations:** 1 Department of Medicine, Division of Gastroenterology, Hepatology and Nutrition, University of Minnesota Medical Center, Minneapolis, Minnesota, USA;; 2 Department of Radiology, University of Minnesota Medical Center, Minneapolis, Minnesota, USA.

**Keywords:** early readmission, quality metric, pancreaticobiliary

## Abstract

**INTRODUCTION:**

Hospital readmission rate is a key hospital metric and represents a substantial burden to patients and the healthcare system. Necrotizing pancreatitis (NP) patients are at high risk of unplanned readmission. The aim of this study was to determine the incidence and predictors of 30-day unplanned readmission after index hospitalization for NP.

**METHODS:**

Adult NP patients who were managed at a single tertiary referral center between 2009 and 2022 were identified from a prospective database and categorized into 2 groups based on 30-day unplanned readmission after index hospitalization. Patients with no follow-up who died during index admission or within 30 days of discharge were excluded. Baseline data on admission including demographic, clinical, interventional, imaging, and discharge characteristics were compared. Multivariable analysis was completed to identify independent predictors of 30-day readmission.

**RESULTS:**

Among 505 patients with NP (male patients—347 [69%], median age—50 years [inter quartile range 37–63]) 191 (37.8%) had at least 1 unplanned readmission. The most common causes of readmission were abdominal pain (40%) and sepsis (27%). On multivariable analysis, independent predictors for early readmission were necrosis collection size ≥ 6 cm (adjusted odds ratio [aOR] 1.91 [1.11–3.30], *P* < 0.03), stay at outside hospital ≥ 14 days before transfer to tertiary center (aOR 2.89 [1.27–6.60], *P* < 0.01), and need for percutaneous feeding tube at the time of discharge (aOR 2.06 [1.01–4.21], *P* < 0.05).

**DISCUSSION:**

Readmission after NP is common and associated with greater mortality at 6 months. Expedited transfer to tertiary center for timely intervention, assiduous follow-up of other high-risk patients (large collections and those who need enteral nutrition) could help avoid readmissions and optimize outcomes.

## Graphical abstract

**Figure:**
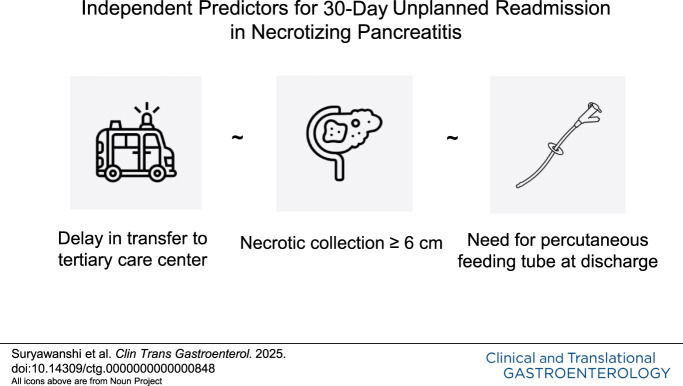

## INTRODUCTION

Acute pancreatitis (AP) is among the most common causes of gastrointestinal hospitalizations in the United States with an estimated annual incidence of 13 to 49 per 100,000 persons (1). Hospital admissions for AP have increased by approximately 30% over the last decade, resulting in $2.6 billion in yearly healthcare costs in the United States (1). Most AP episodes are mild in severity and recover without long-term sequelae, but 10%–20% develop necrosis of pancreatic and/or peripancreatic fat tissue referred to as necrotizing pancreatitis (NP) (2). Necrotic collections often becomes infected, often with accompanying organ failure requiring intensive care admission and prolonged hospital stay, and requiring endoscopic or percutaneous or surgical drainage and necrosectomy (3). A patient's clinical course is often complicated by malnutrition and physical deconditioning, need for enteral or parenteral nutrition, periprocedural complications, and chronic pain often resulting in frequent readmission after discharge.

Hospital readmission rate is an increasingly used quality metric as readmission poses a substantial burden to the patient and the healthcare system (4). Early unplanned readmission (defined as readmission within 30 days after discharge) has been identified as a target for healthcare quality improvement and cost reduction (5,6). The incidence of readmission after AP has been reported to range from 7% to 34% (4). AP patients readmitted within 30 days of discharge have been reported to have a 4.5-fold increased risk of death at 1 year, suggesting that this metric independently affects mortality (7). Although there are robust data regarding AP readmission, there are limited data specifically regarding readmission in NP, with only a single center study showing that 72% of NP patients required readmission (8). Although most readmissions are a consequence of the natural history of the disease, there may be opportunities to optimize clinical outcomes (8). A more detailed understanding the risk factors of readmission is crucial to stratify patients most vulnerable. The aim of this study was to estimate the incidence and etiology for 30-day unplanned readmissions in our well-phenotyped, prospectively maintained cohort after index hospitalization and to identify independent risk factors for readmission.

## METHODS

### Study design and patient population

Consecutive adult patients with NP managed at the University of Minnesota Medical Center between the years 2009–2022 were identified from a prospectively maintained database. Patients who died during index admission or within 30 days of discharge and those who lacked a minimum of 1 year of follow-up data were excluded. In addition, patients who were not managed at our institution for their index NP hospitalization were excluded; however, patients were included if they were transferred to our institution during their index NP hospitalization. Patients with a planned readmission which we defined as any readmission scheduled for planned intervention were excluded. Throughout the duration of the study, all management decisions were made in a multidisciplinary manner involving medical pancreatologists/therapeutic endoscopists, interventional radiologists, intensivists, and surgeons using our previously published algorithm (3,9). This study was approved by the University of Minnesota Institutional Review Board.

### Data collection

The following clinical and radiologic characteristics on initial presentation were collected and analyzed:

Demographics: Age, sex, race, body mass index, etiology of pancreatitis, history of cholecystectomy, and American Society of Anesthesiologists (ASA) class.

Clinical (index admission): length of stay (LOS), transfer from outside hospital (OSH), systemic inflammatory response syndrome (SIRS) on admission, persistent SIRS (SIRS > 48 hours), intensive care unit (ICU) admission, length of ICU stay, single organ failure (acute renal failure, acute respiratory failure, or acute circulatory failure), multiorgan failure, persistent organ failure at 48 hours, NP interventions (endoscopic, percutaneous, or surgical drainage), need for necrosectomy, need for narcotic pain medications during admission, need for narcotics at time of discharge, and need for nutritional support—enteral feeding (nasojejunal/nasogastric/percutaneous endoscopic gastrostomy/jejunostomy) or (total parenteral nutrition) at discharge.

Radiologic: size of necrotic collection, location of necrotic collection (pancreatic, peripancreatic, or both), content of necrotic collection (presence of >30% solid necrosis), presence of splanchnic vein thrombosis (portal vein, superior mesenteric vein, or splenic vein thrombosis), and presence of a disconnected pancreatic duct.

Clinical (readmission): number of readmissions within 30 days of index discharge and etiology of readmission.

### Definitions

SIRS was defined as 2 or more of the following factors: temperature of >38 °C or < 36 °C, heart rate >90 beats per minute, respiratory rate of > 20 breaths per minute or partial pressure of CO_2_ of < 32 mm Hg, and leukocyte count of >12,000 or <4,000 μL or > 10% immature bands. Infected necrosis was defined by the presence of gas in the pancreatic/peripancreatic collection on imaging, and/or positive fluid culture, and/or presence of purulent drainage during endoscopic transluminal drainage (ETD). Organ dysfunction was defined per the modified Marshall scoring system used in the revised Atlanta classification (10). Acute respiratory failure was defined as PaO_2_ of 60 mm Hg despite fraction of inspired oxygen of 25% or need for mechanical ventilation. Acute kidney injury was defined as a serum creatinine level >1.9 mg/dL after rehydration or new need for renal replacement therapy (hemofiltration or hemodialysis). Circulatory failure was defined as systolic blood pressure below 90 mm Hg unresponsive to fluid resuscitation or need for inotropic pressor support.

### Statistical analysis

Baseline demographics, clinical, and imaging characteristics were collected and summarized for all NP patients and compared between those who were readmitted within 30 days and those who were not. Continuous data were reported as median and inter quartile range (IQR), and categorical data were reported as frequency and percentages. Univariate and multivariate analysis was completed to evaluate for the associations between covariates and highlight the predictors for readmission within 30 days. All covariates collected were used in the final multivariable logistic regression model to ensure that we did not miss any meaningful interaction between variables that may affect our predictive outcome. The results were presented as adjusted odds ratios (aORs) for multivariate analysis with 95% confidence intervals. *P* values <0.05 were considered statistically significant. STATA was used for the analysis.

## RESULTS

### Baseline characteristics of study cohort

**Figure 1.**
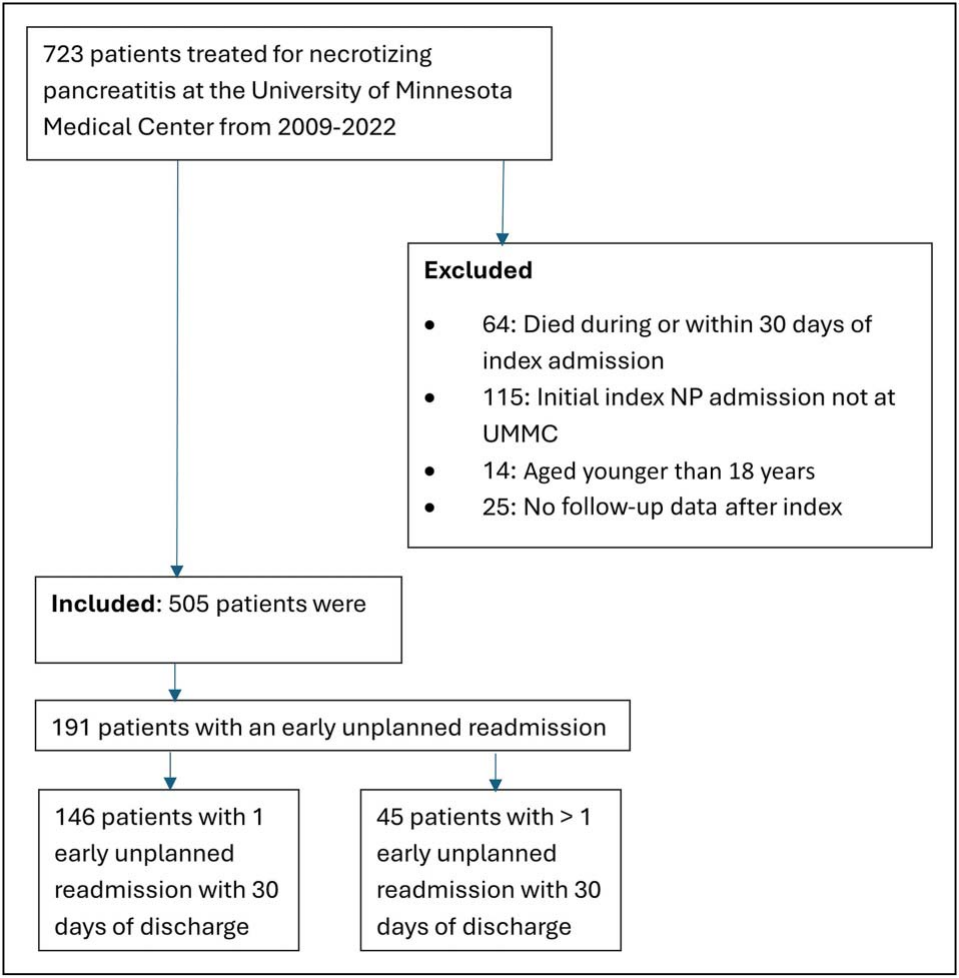
Inclusion and exclusion criteria. NP, necrotizing pancreatitis.

In total, 723 patients with NP managed at our center during the study period were considered for inclusion, 218 were excluded based on predefined criteria (Figure 1). The remaining 505 patients (male patients—347 [69%], median age—50 years [IQR 37–63]) formed the study cohort and were categorized into 2 groups—readmitted within 30 days (i.e., readmission) and those not readmitted (Table 1). Significantly more patients in the early readmission cohort had a longer stay at OSH (>14 days) before transfer 29 (15.2%) vs 27 (8.6%); *P* = 0.02. Demographics including age, race, etiology of pancreatitis, ASA grading and disease severity on admission, and interventions during index hospitalization were similar between the 2 groups. In addition, when studying the imaging characteristics, the extent of pancreatic necrosis, and the location of necrosis (pancreatic, peripancreatic, or both) were also similar between the 2 groups (Table 1). More patients in the early readmission group had necrotic collections >6 cm 137 (75.3%) vs 180 (62.5%); *P* < 0.01. Fewer patients in the early readmission group tolerated per os intake 120 (62.8%) vs 237 (75.5%); *P* < 0.01, and a higher percentage of patients needed enteral nutrition with a nasojejunal tube—36 (18.9%) vs 36 (11.5%); *P* = 0.02 and percutaneous feeding tube—32 (16.8%) vs 35 (11.2%); *P* = 0.07.

**Table 1.** Baseline characteristics

	Readmitted within 30 d N = 191	Non-readmitted N = 314	Pearson χ^2^	*P* value
Demographics, n (%)				
Age, median (IQR)	50 (35–61)	50 (38–64)	2.00	0.16
Sex, male	130 (68.1)	217 (69.1)	0.06	0.81
Race			1.01	0.91
White	166 (86.9)	275 (87.6)		
Black or African American	11 (5.8)	15 (4.8)		
Native American	5 (2.6)	8 (2.6)		
Asian/Pacific Islander	4 (2.1)	10 (3.2)		
Hispanic	5 (2.6)	6 (1.9)		
Etiology of pancreatitis			9.30	0.16
Alcohol	66 (34.6)	124 (39.5)		
Biliary	64 (33.5)	93 (29.6)		
Hypertriglyceridemia	12 (6.3)	12 (3.8)		
Idiopathic	21 (11.0)	55 (17.5)		
Medication	6 (3.1)	6 (1.9)		
Post-ERCP	12 (6.3)	11 (3.5)		
Other	10 (5.2)	13 (4.1)		
History of cholecystectomy	44 (23.0)	70 (22.3)	0.04	0.85
BMI, median (IQR)	28.3 (25.4–33.9)	28.6 (25.0–32.5)	1.87	0.17
ASA grading ≥ 3	114 (59.7)	205 (65.3)	1.60	0.21
BISAP, mean	1.53	1.58	0.22	0.64
Characteristics of disease severity, n (%)				
Transferred from OSH	111 (58.1)	165 (52.6)	1.49	0.22
Length of stay ≥ 14 d at OSH	29 (15.2)	27 (8.6)	5.22	**0.02**
Total length of stay, median in days (IQR)	17 (8–35)	14 (6–28.3)	1.58	0.21
SIRS on admission	135 (70.7)	229 (72.9)	0.30	0.58
Persistent SIRS	96 (50.3)	145 (46.3)	0.74	0.39
Need for ICU admission	57 (29.8)	84 (26.8)	0.56	0.45
*Presence of*				
Multiorgan failure	32 (16.8)	40 (12.7)	1.57	0.21
Persistent organ failure	51 (26.7)	80 (25.5)	0.09	0.76
Interventions during hospitalization, n (%)				
ETD	76 (39.8)	139 (44.3)	0.97	0.32
Percutaneous drainage	49 (25.7)	66 (21.0)	1.45	0.23
Surgical necrosectomy	9 (4.7)	13 (4.1)	0.09	0.76
Endoscopic necrosectomy	56 (29.3)	92 (29.3)	0.00	1.00
Imaging characteristics, n (%)				
Size of necrotic collection ≥ 6 cm^a^	137/182 (75.3)	180/288 (62.5)	8.29	**<0.01**
Extent of pancreatic necrosis, <30%^a^	81/162 (50.0)	139/260 (53.4)	0.48	0.49
Location of necrosis^a^				
Pancreatic	3/184 (1.6)	7/294 (2.4)	0.31	0.58
Peripancreatic	19/184 (10.3)	27/294 (9.2)	0.17	0.68
Both	161/184 (87.5)	260/294 (88.4)	0.09	0.76
*Presence of*				
Disconnected pancreatic duct	57 (29.8)	105 (33.4)	0.71	0.40
Portal vein thrombosis	10 (5.2)	21 (6.7)	0.44	0.51
Superior mesenteric vein thrombosis	8 (4.2)	11 (3.5)	0.15	0.69
Splenic vein thrombosis	20 (10.5)	36 (11.5)	0.12	0.73
Discharge characteristics, n (%)				
Need for percutaneous G or G/J	32 (16.8)	35 (11.2)	3.25	0.07
Need for NJT	36 (18.9)	36 (11.5)	5.30	**0.02**
Need for NGT	2 (1.1)	2 (0.7)	0.25	0.61
Need for TPN	2 (1.1)	0.7 (2.2)	0.95	0.33
Need for narcotics 24 hr prior to discharge	139 (72.8)	239 (76.1)	0.70	0.40
Need for narcotics at time of discharge	130 (68.1)	209 (66.6)	0.12	0.73
Discharge destination, home	145 (75.9)	251 (79.9)	1.13	0.29

ASA, American Society of Anesthesiologists; BISAP; Bedside Index of Severity in Acute Pancreatitis; BMI, body mass index; ERCP, endoscopic retrograde cholangiopancreatography; ETD, endoscopic transluminal drainage; G or GJ, gastrostomy or gastrojejunostomy; ICU, intensive care unit; IQR, interquartile range; NGT, nasogastric tube; NJT, nasojejunal tube; OSH, outside hospital; SIRS, systemic inflammatory response syndrome; TPN, total parenteral nutrition.

Bold entries: were statistically significant with *P* value <0.05. “a” - less patient data available than the listed N.

### Readmission

Among the 505 patients, 191 patients (37.8%) had early readmission and the median time to readmission was 11 days from discharge (IQR 5–18 days). Among the 191 patients who had an early unplanned readmission, 115 (60.2%) were readmitted within 14 days of index hospital discharge. There were 241 total unique readmissions. The most common etiologies for readmission (Table 2) included abdominal pain (N = 96, 39.7%), sepsis (N = 65, 26.8%), gastrointestinal bleeding (N = 12, 5.0%), nausea and vomiting (N = 12, 4.6%), gastric outlet obstruction (N = 10, 4.1%), and feeding tube dysfunction (N = 10, 4.1%). Among the 65 readmissions that were for sepsis, 45 were related to NP and 15 were for sepsis felt likely because of urinary tract infection or pneumonia. 45 patients had more than 1 readmission. Among these 45 patients, there were 95 unique unplanned readmissions with the most common etiologies being abdominal pain (N = 35), sepsis (N = 18), feeding tube dysfunction (N = 9), nausea/vomiting (N = 9), gastrointestinal bleeding (N = 7), and other (N = 17).

**Table 2.** Etiologies of early unplanned readmission

Etiology of early unplanned readmission	% Of total
Abdominal pain (N = 96)	39.7
Sepsis (N = 65)	26.8
Gastrointestinal bleeding (N = 12)	5.0
Nausea and vomiting (N = 11)	4.6
Gastric outlet obstruction (N = 10)	4.1
Feeding tube dysfunction (N = 10)	4.1
Percutaneous drain dysfunction (N = 6)	2.5
Failure to thrive (N = 5)	2.1
Hypoxic respiratory failure (N = 4)	1.7
Alcohol withdrawal (N = 3)	1.3
Fever (N = 2)	0.8
Other (N = 17)	7.1
Status epilepticus	
Kidney stone	
Surgical wound drainage	
Unplanned post procedure monitoring	
Spinal fusion surgery	
Hyponatremia	
Anemia	
Hyperkalemia	
Pulmonary embolism	
Gout flare	
Cholecystitis	
Small bowel obstruction	
Large bowel obstruction	
Hematuria	
Suicidal ideation	
Syncope	
Laparoscopic cholecystectomy	

### Prediction of early readmission in NP

The results of the multivariable logistic regression are summarized in Table 3. Significant independent predictors of 30-day readmission were necrosis collection size ≥6 cm (aOR 1.91 [1.11–3.30], *P* < 0.03), LOS at OSH ≥ 14 days before transfer to tertiary care center (aOR 2.89 [1.27–6.60], *P* < 0.01), and need for percutaneous feeding tube at the time of discharge (aOR 2.06 [1.01–4.21], *P* < 0.05).

**Table 3.** Multivariable analysis between readmitted patients vs non-readmitted patients

	aOR	95% CI lower	95% CI upper	*P* value
Age	0.99	0.98	1.01	0.38
Sex, male	0.97	0.61	1.56	0.90
Race, white	1.08	0.55	2.12	0.83
Etiology of pancreatitis, alcohol	0.75	0.46	1.22	0.25
History of cholecystectomy	0.95	0.56	1.59	0.83
BMI	1.02	0.99	1.06	0.20
ASA grading ≥ 3	0.64	0.38	1.08	0.09
Transferred from OSH	1.09	0.67	1.77	0.72
Length of stay ≥ 14 d at OSH	2.89	1.27	6.60	<0.01
Total length of stay	0.99	0.97	1.01	0.18
SIRS on admission	0.79	0.43	1.44	0.44
Persistent SIRS	1.27	0.69	2.32	0.44
Need for ICU admission	1.11	0.55	2.27	0.77
*Presence of*				
Multiorgan failure	1.26	0.68	2.35	0.46
Persistent organ failure	0.77	0.40	1.48	0.43
ETD	0.73	0.39	1.36	0.32
Percutaneous drainage	1.22	0.68	2.20	0.50
Surgical necrosectomy	0.83	0.24	2.86	0.76
Endoscopic necrosectomy	0.95	0.47	1.92	0.88
Size of necrotic collection ≥ 6 cm	1.91	1.11	3.30	<0.03
Extent of pancreatic necrosis, <30%	0.72	0.44	1.18	0.20
Location of necrosis				
Pancreatic	0	0		1.0
Peripancreatic	0	0		1.0
Both	0	0		1.0
*Presence of*				
Disconnected pancreatic duct	0.96	0.60	1.54	0.88
Portal vein thrombosis	0.51	0.20	1.32	0.17
Superior mesenteric vein thrombosis	1.53	0.51	4.57	0.45
Splenic vein thrombosis	0.78	0.40	1.53	0.48
Need for percutaneous G or G/J tube	2.06	1.01	4.21	<0.05
Need for NJT	1.38	0.70	2.71	0.35
Need for NGT	2.36	0.18	30.94	0.51
Need for TPN	0.29	0.03	2.87	0.29
Need for narcotics 24 hr prior to discharge	0.62	0.25	1.51	0.29
Need for narcotics at time of discharge	1.20	0.53	2.74	0.66
Discharge destination, home	0.95	0.47	1.91	0.88

aOR, adjusted odds ratio; ASA, American Society of Anesthesiologists; BMI, body mass index; ETD, endoscopic transluminal drainage; G or GJ, gastrostomy or gastrojejunostomy; ICU, intensive care unit; IQR, interquartile range; LOS, length of stay; NGT, nasogastric tube; NJT, nasojejunal tube; OSH, outside hospital; SIRS, systemic inflammatory response syndrome; TPN, total parenteral nutrition.

*P* value <0.05 considered significant.

Further exploratory subgroup analysis of independent predictors was performed. Patients with LOS at OSH ≥ 14 days before transfer to tertiary care center had a greater initial illness severity with a higher incidence of persistent SIRS (70% vs 45%, *P* < 0.001), multiorgan failure (25% vs 13%, *P* < 0.02), and need for ICU admission (45% vs 26%, *P* < 0.01). They needed more interventions ETD (70% vs 39%, *P* < 0.001), endoscopic necrosectomy (35% vs 25%, *P* < 0.001), and percutaneous drainage (38% vs 21%, *P* < 0.01) on transfer.

### Impact of readmission in short-term mortality following NP

The all-cause mortality in our NP cohort was 57 (11.3%) after index hospitalization. Patients in the early readmission arm were at increased risk of medium-term mortality at 6 months (28.6% vs 5.8%, *P* = 0.016). A multivariable analysis after accounting for comorbidities and severity showed that 30-day readmission was an independent predictor for mortality at 6 months (aOR 6.11 [1.01–36.9], *P* < 0.05) (Supplemental Table, http://links.lww.com/CTG/B317). However, early readmission did not increase long-term mortality at 1 year (33% vs 25%, *P* = 0.5).

## DISCUSSION

In a cohort of 505 patients with NP, 37.8% of patients were readmitted within 30 days after initial hospitalization. Most early unplanned readmissions were due to abdominal pain and sepsis. Readmitted patients were independently at increased risk of mortality at 6 months. The strongest predictors for readmission included larger necrotic collections (>6 cm), need for enteral nutrition through percutaneous feeding tube at discharge, and delay in transfer to a tertiary care facility. The findings of this study suggest that patients most vulnerable for early readmission can be readily identified, offering an opportunity to optimize clinical outcomes. Patients identified as high risk of readmission may be scheduled for earlier outpatient follow-up to improve overall care and reduce healthcare costs (11).

The previously reported rate of unplanned readmission in AP ranges from 7 to 34%, and severity of pancreatitis affects readmission rates (4). Readmission rates after severe AP range from 20 to 75% in population-based studies, representing the highest among pancreaticobiliary conditions (4,8). The only other study evaluating consecutive NP patients showed an early readmission rate of 28%, which was lower than found in our study (12). Our study further validates that NP carries a higher risk of early readmission after index hospitalization (4,12,13).

Early readmission has been explored as a surrogate for mortality in other conditions such as congestive heart failure, wherein 30-day readmission was a risk factor of long-term mortality independent of severity of heart failure and comorbidities (14,15). It has been proposed that a sustained inflammatory milieu from NP may exert unfavorable effects on or accelerate preexisting comorbid illnesses (7). In our study, NP patients readmitted within 30 days were 6 times more likely to die within 6 months when compared with those who were not, suggesting that this quality metric can independently affect medium-term mortality. This corroborates the findings of an earlier study which showed a 4.5-fold increased risk of mortality in readmitted AP patients further highlighting the impact of this metric in clinical outcomes (4,7).

Most of the early readmissions in our study were due to symptomatic necrosis including abdominal pain, nausea/vomiting, and nearly 20% were due to infected necrosis. Abdominal pain accounted for nearly 40% of the readmissions, as in other studies (6,8,12). Although optimizing abdominal pain before discharge and timely outpatient follow-up are vital to prevent readmission, a recent study showed that titration of narcotics to pain severity is unlikely to affect readmission rate (12). In our study, narcotic medications during the predischarge and postdischarge period did not affect early readmission rate. Nausea and vomiting accounted for a lower proportion of early readmissions in our study. Antiemetic prescriptions and prescriptions for outpatient fluid infusions may have resulted in fewer early readmissions. While a substantial proportion of the early readmissions were due to the inherent nature of the disease process and may not be imminently preventable, some concerted efforts can reduce readmission. These include dedicated management by an inpatient pancreas consult team to ensure meticulous coordination, provide continuous patient and family education about the complex disease process, and involve registered dietitians. Such a specialized team also ensures discharge planning for seamless transition to outpatient care and planning early scheduled necrosectomy after endoscopic drainage instead of on demand necrosectomy to reduce readmission from infected necrosis. Once discharged, a dedicated pancreas nurse coordinator serves as primary point of contact to patient and families for personalized continuity of care throughout the protracted course of NP. Such a strategy for improved outpatient communication was shown to identify treatable problems such as feeding tube dysfunction, percutaneous drain dysfunction, and failure to thrive and significantly reduce rate of readmission from 64% to 45% in a recent study (16).

Among the predictors for early readmission, patients with larger necrotic collections (>6 cm) were twice as likely to require early readmission. Larger necrotic collections are less likely to spontaneously resolve without intervention, with higher risk of eventual symptoms and need for intervention and readmission. Our group previously established that when patients with 6 cm or larger necrotic collection were discharged without intervention in their index hospitalization, they were at 8 times likely to undergo future drainage or necrosectomy after discharge (17). Another study from Mayo clinic showed that necrotic collections > 10 cm independently predicted need for additional step up therapies after the index drainage procedure including endoscopic necrosectomy, adjuvant drainage, or surgical intervention (18). In our study, on readmission, patients with 6 cm or larger necrotic collections underwent more percutaneous and/or endoscopic drainage/necrosectomy, further underscoring the need for early scheduled intervention on discharge to avoid readmission. One area of further inquiry in NP patients is understanding how urgently or at what frequency to bring back patients for initial or repeat necrosectomy. One multicenter study from the US Pancreatitis Study Group showed that an approach incorporating upfront necrosectomy at the index endoscopic intervention vs a traditional step-up approach could reduce the number of reinterventions required to achieve treatment success. This study showed that there was no difference in all-cause hospital readmissions at 6 months between the 2 treatment groups; however, they did not look at rates of early unplanned readmission at 30 days and only included patients with infected necrosis, highlighting a potential area for further study (19).

Patients discharged on enteral nutrition were at increased risk of early readmission (aOR 1.63 (1.06–2.51), *P* = 0.03) with a major reason being failure to thrive from tube dysfunction. Discharging a patient on less than a solid diet suggests that an underlying process may be still actively evolving and was one of the strongest predictors of early readmission in an earlier study (OR 23.8; 95% CI 4.8–118.2) (6). Inability to tolerate oral diet is also a component of the externally validated Pancreatitis Activity Scoring System, and Pancreatitis Activity Scoring System > 60 was associated with early readmission (OR 5.0 [2.4, 10.7]) (20,21). Among reasons for early readmission, failure to thrive from tube dysfunction accounted for 7.5%, but they are imminently preventable with timely outpatient reassessment.

Prolonged LOS at an OSH has been considered as a surrogate for a delay in interhospital transfer with implications for patient outcomes in the surgical and critical care literature (22). Delay in interhospital transfer has been associated with increased mortality and longer length of hospital stay in patients with surgical sepsis. Given the greater illness severity on arrival, these patients may be best served in tertiary care center (22). In our study, NP patients with a prolonged LOS at outside facility (>14 days) likely experienced interhospital transfer delay and were twice likely to have early readmission. These patients had a greater initial illness severity with higher incidence of persistent SIRS, persistent organ failure, and need for ICU admission at the outside facility. They needed more interventions ETD, endoscopic necrosectomy, and percutaneous drainage on transfer. Prehospital triage system should therefore prioritize and expedite the transfer of NP patients to tertiary care facilities to optimize outcomes.

Although several studies evaluating AP of any etiology and severity reported no differences in readmission rate based on comorbidities, comorbidities have also been showing to increase hospital readmission rates (4). In our cohort, ASA ≥ 3 patients needed more ICU admission, underwent more intervention for NP and as a result had significantly longer LOS. Given the greater severity of illness, they were less likely to be discharged and hence have less readmissions.

The strengths of this study include use of a prospectively maintained database of all consecutive patients admitted to the hospital with NP of any extent and inclusion of patients managed by the entire spectrum of endoscopic, percutaneous, and surgical interventions available for NP, baseline assessment of severity by means of validated scores, and defined radiological follow-up. Our cohort was substantial for a single-center study and was performed in a large tertiary center with significant pancreaticobiliary expertise.

Limitations of our study included reporting from a single center. A sizeable proportion of patients were referred from out of state, and we may not have adequately captured all the readmission data given our incomplete access to electronic health records. However, we believe that this will not significantly affect our estimates as most referring hospitals tend to transfer these patients back to our center given their complex nature of management. The cause for readmission may be multifactorial, but the single most important reason was included for analysis.

In conclusion, early unplanned readmission is common among NP and is at increased risk of mortality at 6 months. Our study findings may guide the identification of the highest risk patients who will benefit from more intensive outpatient follow-up.

## CONFLICTS OF INTEREST

**Guarantor of the article:** Guru Trikudanathan, MD.

**Specific author contributions:** G.S.: involved in data collection, data analysis, manuscript writing and review. M.G.: involved in data analysis. N.F.: involved in data collection. S.K.A., N.A., S.M., M.F.: involved in manuscript writing and review. G.T.: study supervisor.

**Financial support:** None to report.

**Potential competing interests:** S.K.A., N.A., S.M., and G.T. are consultants for Boston Scientific Corporation. G.S., M.G., N.F., and M.L.F. declare no conflicts of interest.

**Study Highlights**

**WHAT IS KNOWN**

✓ Readmission is a key hospital metric.
✓ Necrotizing patients are at high risk of readmission.

**WHAT IS NEW HERE**

✓ Predictors of early readmission include delayed transfer, need for enteral nutrition, and large necrotic collections.

## Supplementary Material

## ABBREVIATIONS:

NP: necrotizing pancreatitisa
aOR: adjusted odds ratio
AP: acute pancreatitis
ASA: American Society of Anesthesiologists
ICU: intensive care unit
SIRS: systemic inflammatory response syndrome
ETD: endoscopic transluminal drainage
IQR: interquartile range
BISAP: bedside index of severity in acute pancreatitis
OSH: outside hospital
BMI: body mass index
NJT: nasojejunal tube
NGT: nasogastric Tube
TPN: total parenteral nutrition
LOS: length of stay
PO: per os
UMMC: University of Minnesota Medical Center
