# Supplementary material for: Predictors of 30-Day Unplanned Readmission in Necrotizing Pancreatitis: A 12-Year Experience From a Tertiary Care Center
Source: Clin Transl Gastroenterol. 2025 May 23;16(7):e00848. doi: 10.14309/ctg.0000000000000848 (PMC12330361; doi:10.14309/ctg.0000000000000848)
Supplement: Supplementary file 1 [file ct9-16-e00848-s001.docx]

|  | **Univariate analysis (Cohort)** | | | | **Multivariable analysis (Cohort)** | | | |
| --- | --- | --- | --- | --- | --- | --- | --- | --- |
| *Characteristic* | **OR** | **95% CI Lower** | **95% CI Upper** | **P-value** | **aOR** | **95% CI Lower** | **95% CI Upper** | **P-value** |
| ASA ≥ 3 | 0.87 | 0.15 | 4.92 | 0.87 | 0.72 | 0.11 | 4.91 | 0.74 |
| Obesity | 1.23 | 0.27 | 5.48 | 0.79 | 1.05 | 0.20 | 5.47 | 0.95 |
| Transfer from OSH | 2.07 | 0.38 | 11.31 | 0.39 | 1.06 | 0.14 | 8.29 | 0.96 |
| Persistent multiorgan failure at admission | 2.42 | 0.52 | 11.30 | 0.25 | 1.51 | 0.24 | 9.43 | 0.66 |
| Readmission within 30 days of discharge | 6.8 | 1.23 | 37.66 | **0.02** | 6.11 | 1.01 | 36.87 | **<0.05** |

Supplemental Table 1: Univariable and multivariable analysis looking at mortality at 6 months. OSH (Outside Hospital), ASA (American Society of Anesthesiologists), aOR (Adjusted Odds Ratio), OR (Odds Ratio). *P-value <0.05 considered significant.*
